# CRISPR/Cas9-mediated gene knockout in the mouse brain using *in utero* electroporation

**DOI:** 10.1038/srep20611

**Published:** 2016-02-09

**Authors:** Yohei Shinmyo, Satoshi Tanaka, Shinichi Tsunoda, Kazuyoshi Hosomichi, Atsushi Tajima, Hiroshi Kawasaki

**Affiliations:** 1 Department of Biophysical Genetics, Graduate School of Medical Sciences, Kanazawa University, Ishikawa 920-8640, Japan; 2 Brain/Liver Interface Medicine Research Center, Kanazawa University, Ishikawa 920-8640, Japan; 3 Department of Bioinformatics and Genomics, Graduate School of Medical Sciences, Kanazawa University, Ishikawa 920-8640, Japan

## Abstract

The CRISPR/Cas9 system has recently been adapted for generating knockout mice to investigate physiological functions and pathological mechanisms. Here, we report a highly efficient procedure for brain-specific disruption of genes of interest *in vivo*. We constructed pX330 plasmids expressing humanized Cas9 and single-guide RNAs (sgRNAs) against the *Satb2* gene, which encodes an AT-rich DNA-binding transcription factor and is responsible for callosal axon projections in the developing mouse brain. We first confirmed that these constructs efficiently induced double-strand breaks (DSBs) in target sites of exogenous plasmids both *in vitro* and *in vivo*. We then found that the introduction of pX330-Satb2 into the developing mouse brain using *in utero* electroporation led to a dramatic reduction of Satb2 expression in the transfected cerebral cortex, suggesting DSBs had occurred in the *Satb2* gene with high efficiency. Furthermore, we found that Cas9-mediated targeting of the *Satb2* gene induced abnormalities in axonal projection patterns, which is consistent with the phenotypes previously observed in *Satb2* mutant mice. Introduction of pX330-NeuN using our procedure also resulted in the efficient disruption of the *NeuN* gene. Thus, our procedure combining the CRISPR/Cas9 system and *in utero* electroporation is an effective and rapid approach to achieve brain-specific gene knockout *in vivo*.

Technologies for disrupting the genome in living animals are essential for elucidating the physiological mechanisms of our body and the pathological mechanisms of diseases, and for contributing to the discovery of new therapeutic interventions for human diseases. One technique which has been widely utilized is homologous recombination-based gene targeting in embryonic stem (ES) cells^1^. In addition, conditional mutagenesis using ES cells that relies on the DNA recombinase Cre and its recognition site loxP has become a valuable tool to achieve gene targeting in selected cell types^2^. Although this gene targeting technology using ES cells is extensively utilized for generating various kinds of genetically modified mice, it is relatively arduous, costly and time-consuming.

Recently, a simple and efficient genome targeting technology has been developed based on the microbial type II clustered, regularly interspaced, short palindromic repeats (CRISPR)/associated protein (Cas) adaptive immune systems derived from *Streptococcus pyogenes*^3^. Cas9 endonuclease guided by a duplex of mature CRISPR RNA (crRNA) and transactivating crRNA (tracrRNA) cleaves trespassing DNA from bacteriophages or plasmids in a sequence-specific manner^3^. In mammalian cells, it has been demonstrated that a combination of Cas9 and sgRNA, which is an artificial chimera of crRNA and tracrRNA, caused site-specific DSBs, and as a result, genome targeting via insertions and deletions (indels) was caused by error-prone non-homologous end-joining (NHEJ)^4^,^5^. These features of the CRISPR/Cas9 system facilitated the generation of mice carrying mutations in specific genes^6^. Indeed, co-injection of Cas9 mRNA and sgRNAs targeting the Ten-eleven translocation (Tet) family members *Tet1* and *Tet2* into zygotes resulted in the efficient and rapid generation of mice with biallelic mutations in both *Tet1* and *Tet2* genes^6^.

Achieving organ-specific gene knockout using the CRISPR/Cas9 system has been an important challenge because if a gene is knocked out throughout the body, it often leads to embryonic lethality. By combining the CRISPR/Cas9 system and *in utero* electroporation, which is a recently invented, rapid and efficient technique to deliver transgenes into the living rodent brain^7^,^8^,^9^,^10^,^11^,^12^, here we report a brain-specific gene knockout method, and demonstrate the power of our simple electroporation-based gene knockout in the living mouse brain.

## Results

### Construction of CRISPR/Cas9 plasmids targeting the *Satb2* gene with pX330

To examine the effectiveness of gene knockout in the developing mouse cortex by combining the CRISPR/Cas9 system and *in utero* electroporation, we used the transcription factor Satb2. Satb2 is expressed in post-mitotic neurons in the cerebral cortex of the developing mouse brain, and is required for sending callosal axons to the other side of the brain^13^,^14^,^15^. Indeed, the axons derived from *Satb2*-deficient neurons are unable to cross the corpus callosum and instead project subcortically. Thus, in addition to loss of Satb2 expression, these altered axonal projections can be used to assess whether the *Satb2* gene has been disrupted.

**Figure 1:**
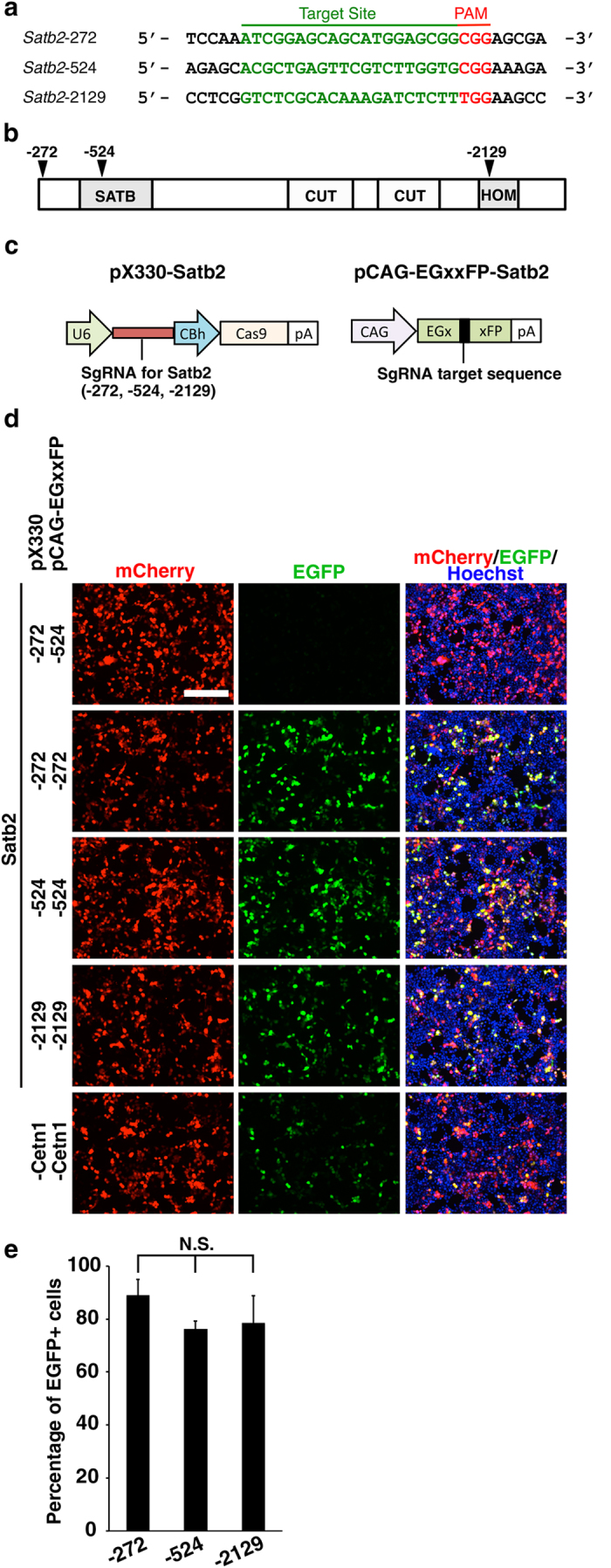
Construction and validation of CRISPR/Cas9 plasmids for Satb2 in HEK293T cells. (**a**) Three different target sites (green) followed by the PAM sequence (red) in the *Satb2* gene. (**b**) Schematic representation of the domain structure of Satb2. Arrowheads indicate the three sgRNA target sites used here. The anti-Satb2 antibody used in Figs 3 and 4 recognizes the C-terminal region of the Satb2 protein. (**c**) pX330-Satb2 plasmid and pCAG-EGxxFP-Satb2 target plasmid. pX330-Satb2 contains expression cassettes of humanized Cas9 and sgRNA for Satb2. pCAG-EGxxFP-Satb2 contains a genomic fragment including the sgRNA target sequence (black) between 5′ and 3′ EGFP fragments (green). (**d**) The effects of three kinds of pX330-Satb2 on EGFP expression derived from pCAG-EGxxFP-Satb2 target plasmids. pX330-Satb2, pCAG-EGxxFP-Satb2 and pCAG-mCherry were co-transfected into HEK293T cells. When pCAG-EGxxFP-Satb2 contained appropriate target sequences, HEK293T cells transfected with pX330-Satb2-272, -524 or -2129 exhibited EGFP signal in the majority of mCherry-positive transfected cells. pX330-Cetn1 and pCAG-EGxxFP-Cetn1 were used as positive controls. Scale bar = 200 μm. (**e**) The percentages of mCherry-positive transfected cells which became EGFP-positive. HEK293  cells were transfected with pX330-Satb2, pCAG-EGxxFP-Satb2 and pCAG-mCherry. N.S., not significant; one-way analysis of variance (*n* = 4 independent experiments). Error bars indicate SD.

To select Cas9 target sites in the *Satb2* gene, we searched for 20-nucleotide sequences followed by the protospacer-adjacent motif (PAM) sequence (NGG) after the translational start site (ATG). We used the CRISPR design tool (http://crispr.mit.edu/) to minimize off-targeting effects and chose three target sites, *Satb2*-272, *Satb2*-524 and *Satb2*-2129, which correspond to exons coding for the N-terminal region, the SATB domain and the homeodomain, respectively (Fig. 1a,b)^16^. For the construction of CRISPR/Cas9 plasmids, we chose the pX330 plasmid, which is commonly injected into the pronuclei of fertilized eggs to generate mutant mice^17^,^18^. The *Satb2* target sequences were cloned into the pX330 plasmid, in which humanized Cas9 and sgRNA are simultaneously expressed under the chicken beta-actin hybrid (CBh) and human U6 promoters, respectively (Fig. 1c, left)^4^.

### Validation of the effects of pX330-Satb2 plasmids *in vitro* and *in vivo*

To evaluate the effectiveness of three plasmids, pX330-Satb2-272, -524 and -2129, we used the pCAG-EGxxFP target plasmid as a reporter plasmid, which contains the CAG promoter plus 5′ and 3′ EGFP fragments that share 482 bp (Fig. 1c, right)^17^,^18^. When gene-targeted DSBs are induced, this plasmid produces EGFP reconstituted by homology-dependent repair (HDR). *Satb2* genomic fragments containing sgRNA target sites were inserted into the multi-cloning site of the pCAG-EGxxFP plasmid (pCAG-EGxxFP-Satb2).

We first co-transfected pX330-Satb2 and pCAG-EGxxFP-Satb2 into HEK293T cells with pCAG-mCherry to label transfected cells, and fluorescence signals derived from mCherry and reconstituted EGFP were observed 48 hours later. We observed no EGFP fluorescence in negative control samples, in which pX330-Satb2 and pCAG-EGxxFP-Satb2 contained different sequences (Fig. 1d, pX330-Satb2-272 and pCAG-EGxxFP-Satb2-524). In contrast, when pCAG-EGxxFP-Satb2 reporter plasmids contained appropriate target sequences, HEK293T cells transfected with pX330-Satb2-272, -524 or -2129 exhibited EGFP signal in the majority of mCherry-positive cells (Fig. 1d). It should be noted that EGFP signals in these cells were stronger than those of HEK293T cells transfected with the pCAG-EGxxFP-Centrin1 (Cetn1) and pX330-Cetn1 plasmids (Fig. 1d), which are commonly used as a positive control of the CRISPR/Cas9 system^18^.

We then quantified the number of EGFP-positive cells induced by the pX330-Satb2 constructs. We found that 89%, 76% and 79% of pX330-Satb2-272-, pX330-Satb2-524- and pX330-Satb2-2129-transfected cells became EGFP-positive, respectively (Fig. 1e). The differences in the percentage of EGFP-positive cells were not statistically significant. These results suggest that pX330-Satb2-272, -524 and -2129 effectively induce DSBs in targeted sites of reporter plasmids in HEK293T cells.

**Figure 2:**
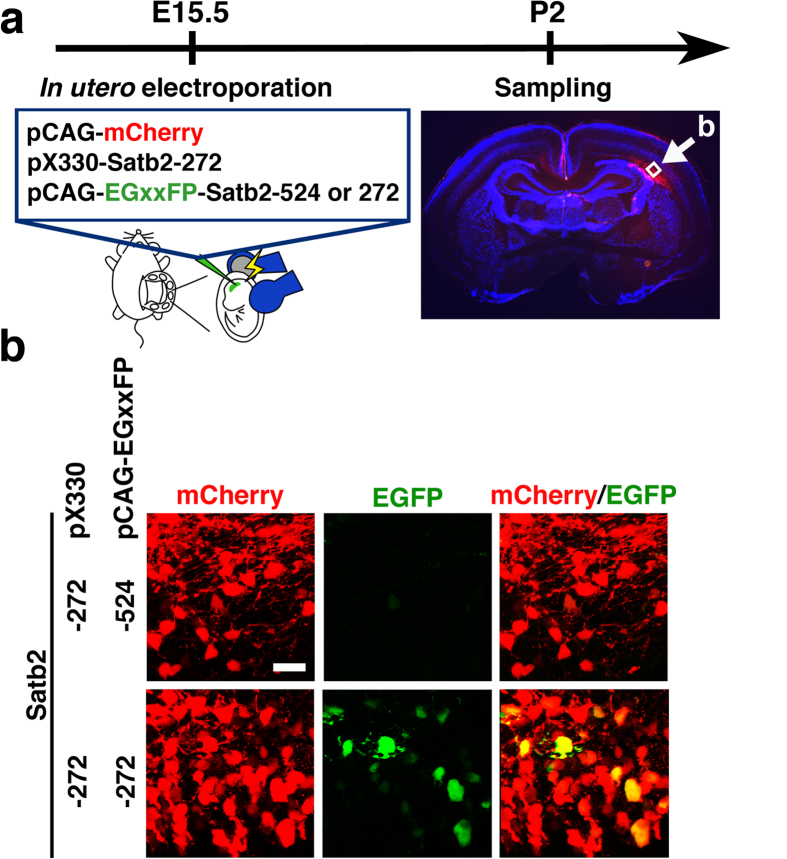
Validation of the effects of pX330-Satb2 plasmids on pCAG-EGxxFP-Satb2 target plasmid in the mouse brain. (**a**) Experimental procedure. Cortical neurons were co-transfected with pCAG-mCherry, pX330-Satb2-272 plus either pCAG-EGxxFP-Satb2-272 or pCAG-EGxxFP-Satb2–524 using *in utero* electroporation at E15.5, and coronal sections were prepared at P2. The white square indicates the region which was magnified and shown in (**b**). (**b**) High magnification confocal microscopic images. Note that EGFP signal was observed in mCherry-positive neurons transfected with pX330-Satb2-272 and pCAG-EGxxFP-Satb2-272, which contained appropriate target sequences (lower panels). In contrast, when pCAG-EGxxFP-Satb2-524, which contained different target sequences, was used as a reporter plasmid, EGFP signals were not observed (upper panels). Scale bar = 20 μm.

To examine whether pX330-Satb2 plasmids could induce gene-targeted DSBs in the developing mouse cortex, we introduced pCAG-mCherry, pX330-Satb2-272 and pCAG-EGxxFP-Satb2-272 into the developing mouse brain using *in utero* electroporation at embryonic day 15.5 (E15.5) (Fig. 2a), which resulted in gene expression in layer 2/3 neurons of the cerebral cortex. We prepared sections of the cerebral cortex at postnatal day 2 (P2) and observed EGFP fluorescence in mCherry-positive transfected cells in the ventricular zone (VZ) and subventricular zone (SVZ) (Fig. 2b, lower panels). In contrast, when pX330-Satb2-272 and pCAG-EGxxFP-Satb2-524 were introduced, no EGFP signals were observed (Fig. 2b, upper panels). These results suggest that pX330-Satb2 plasmids induce DSBs in targeted sites of pCAG-EGxxFP-Satb2 plasmids in the living mouse brain.

### pX330-Satb2 plasmids effectively suppressed endogenous Satb2 expression in the mouse brain

**Figure 3:**
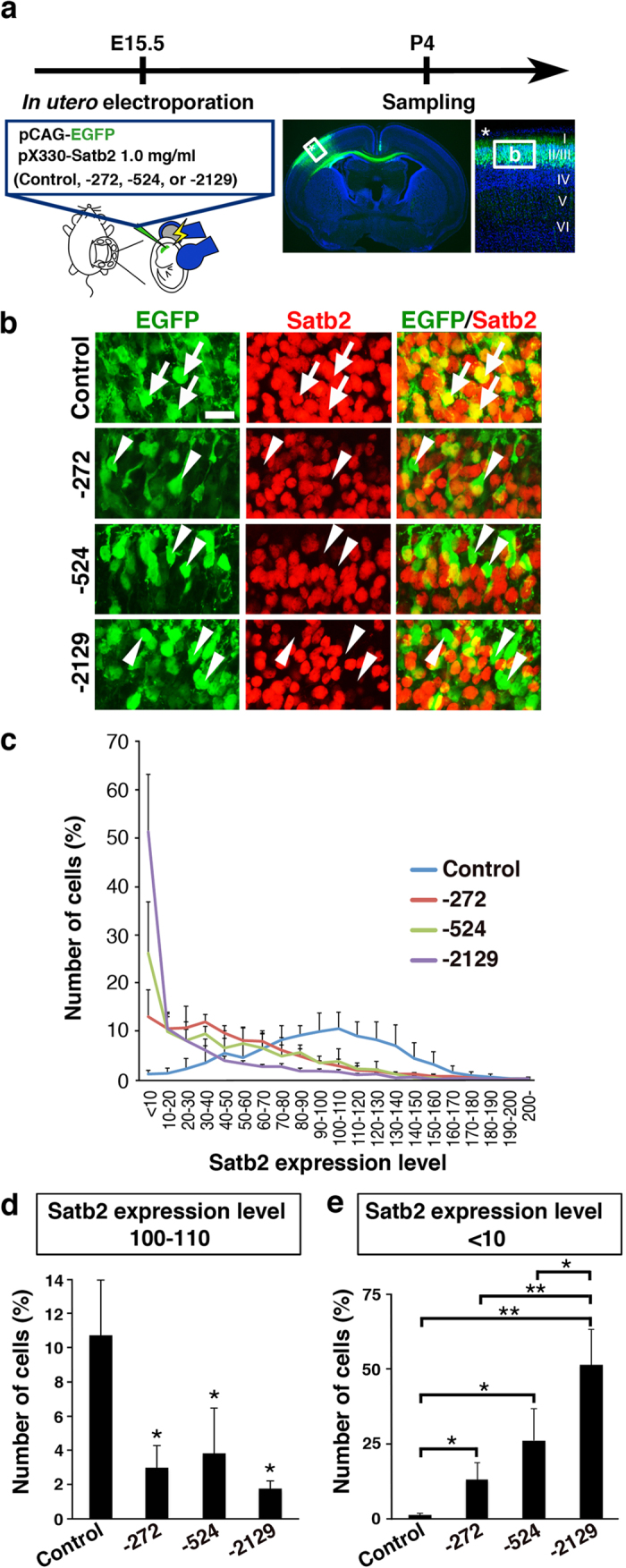
pX330-Satb2 effectively eliminates endogenous Satb2 expression in the mouse cortex. (**a**) Experimental procedure. Layer 2/3 neurons were co-transfected with pCAG-EGFP and pX330-Satb2 (1.0 mg/ml) using *in utero* electroporation at E15.5. Coronal sections were prepared at P4 and stained with anti-Satb2 antibody (red), anti-EGFP antibody (green) and Hoechst 33342 (blue). The area in the box (asterisk) of the middle panel was magnified and shown in the right panel. The box in the right panel is used to indicate the region of each sample which was magnified and shown in (**b**). Numbers indicate the corresponding layers in the cerebral cortex. (**b**) High magnification confocal images of layer 2/3 neurons. Arrows indicate normal Satb2 expression in EGFP-positive neurons transfected with pX330 control plasmid. Arrowheads indicate a dramatic reduction of Satb2 expression in EGFP-positive neurons transfected with pX330-Satb2-272, -524 or -2129. Scale bar = 20 μm. (**c**) Histogram of the expression levels of Satb2 in transfected neurons. The average Satb2 signal intensity in EGFP-positive neurons transfected with a pX330 control vector was set as Satb2 expression level 100. The number of neurons with Satb2 expression level 100-110 and the number of neurons with Satb2 expression level <10 were also shown in (d) and (e), respectively. (**d**) The number of neurons expressing normal levels of Satb2 protein was greatly reduced by pX330-Satb2-272, -524 and -2129. (**e**) The number of neurons with no Satb2 expression was markedly increased by pX330-Satb2-272, -524 and -2129. Note that the number of neurons with no Satb2 expression was significantly larger among neurons transfected with pX330-Satb2-2129 compared with those with pX330-Satb2-272 or -524. *p < 0.05; **p < 0.01; Welch’s *t*-test (*n* = 4 pups for each condition). Error bars indicate SD.

To knock out endogenous *Satb2* in the mouse brain, we co-transfected 0.5 mg/ml pCAG-EGFP and 1.0 mg/ml pX330-Satb2 into layer 2/3 neurons of the mouse cerebral cortex using *in utero* electroporation at E15.5 (Fig. 3a). We chose layer 2/3 neurons because endogenous Satb2 is preferentially expressed in layer 2/3 of the developing mouse cerebral cortex^13^,^14^,^15^. We prepared coronal sections at P4 and examined the expression of Satb2 protein using immunostaining. When the pX330 control plasmid was transfected, Satb2 expression was observed in almost all EGFP-positive transfected layer 2/3 neurons (Fig. 3b, arrows). In contrast, we found that Satb2 expression was markedly suppressed in EGFP-positive neurons when transfected with pX330-Satb2-272, -524 or -2129 (Fig. 3b, arrowheads). To quantify these effects, we measured Satb2 immunoreactivity in EGFP-positive neurons (Fig. 3c). We found that the number of neurons expressing normal levels of Satb2 protein was greatly reduced by pX330-Satb2-272, -524 and -2129 (Fig. 3c,d). Furthermore, we also found that the number of neurons with no Satb2 expression was markedly increased by pX330-Satb2-272, -524 and -2129 (Fig. 3c,e). The number of neurons with no Satb2 expression was significantly larger among neurons transfected with pX330-Satb2-2129 compared with those with pX330-Satb2-272 or -524 (Fig. 3e), suggesting that pX330-Satb2-2129 is most effective in disrupting the *Satb2* gene.

**Figure 4:**
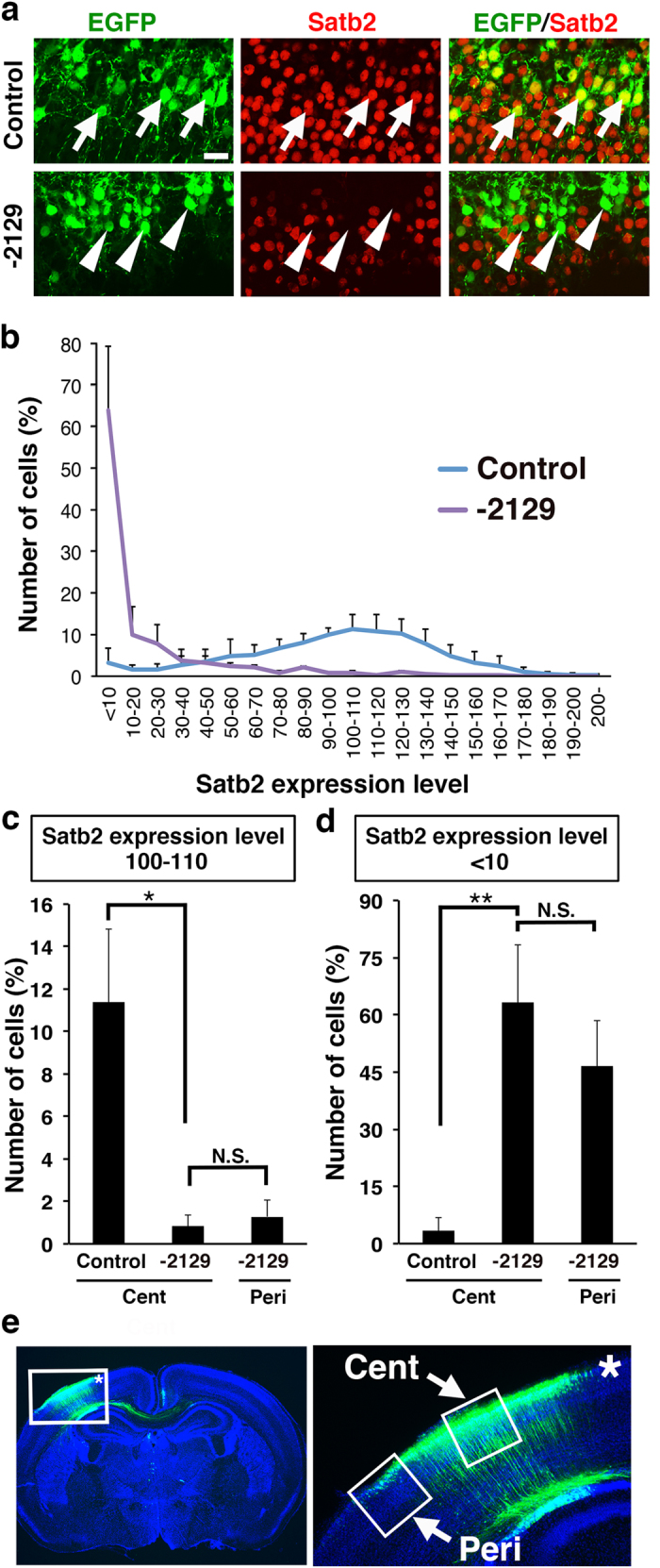
Higher concentration of pX330-Satb2-2129 efficiently disrupts the *Satb2* gene. (**a**) Layer 2/3 neurons were co-transfected with pCAG-EGFP and pX330-Satb2 (2.5 mg/ml) using *in utero* electroporation at E15.5. Coronal sections were prepared at P4 and immunostained with anti-Satb2 antibody (red), anti-EGFP antibody (green) and Hoechst 33342 (blue). Arrows indicate normal Satb2 expression in EGFP-positive neurons transfected with a pX330 control plasmid. Arrowheads indicate a dramatic reduction of Satb2 expression in EGFP-positive neurons transfected with pX330-Satb2-2129. Scale bar = 20 μm. (**b**) Histogram of the expression levels of Satb2 in transfected neurons. The average Satb2 signal intensity in EGFP-positive neurons transfected with a pX330 control vector was set as Satb2 expression level 100. The number of neurons with Satb2 expression level 100-110 and that of neurons with Satb2 expression level <10 were also shown in (**c**) and (**d**), respectively. (**c**) The number of neurons with normal levels of Satb2 expression was markedly reduced by pX330-Satb2-2129 in the central (Cent) and peripheral (Peri) regions of the EGFP-positive cortical area. (**d**) The number of neurons with no Satb2 expression was greatly increased by pX330-Satb2-2129 in the central and peripheral regions of the EGFP-positive cortical area. *p < 0.05; **p < 0.01; N.S., not significant; Welch’s *t*-test (*n* = 4 pups for each condition). Error bars indicate SD. (**e**) A coronal section showing central (Cent) and peripheral (Peri) regions of the EGFP-positive cortical area used in (**c,d**). The area inside the box (asterisk) in the left panel was magnified and shown in the right panel.

To examine if higher concentrations of pX330-Satb2-2129 more efficiently disrupt the *Satb2* gene, we co-transfected layer 2/3 neurons with 0.5 mg/ml pCAG-EGFP and a high concentration of pX330-Satb2-2129 (2.5 mg/ml) using *in utero* electroporation at E15.5. Satb2 immunoreactivity was strongly suppressed in the majority of EGFP-positive transfected neurons (Fig. 4a, arrowheads). A quantitative analysis revealed that, in the central region of the transfected cortical area (Fig. 4e, Cent), Satb2 expression was lost in 64% of pX330-Satb2-2129-transfected neurons (Fig. 4b,d), and that the number of neurons with normal levels of Satb2 expression was markedly reduced by pX330-Satb2-2129 (Fig. 4b,c). These results suggest that higher concentrations of pX330-Satb2-2129 induce disruption of the *Satb2* gene more efficiently.

We next compared the efficiency of Satb2 knockout in the peripheral (Fig. 4e, Peri) and the central regions (Fig. 4e, Cent) of the EGFP-positive cortical area, and found that Satb2 expression was strongly suppressed in both regions (Fig. 4c,d). Although the suppression tended to be weaker in the peripheral region than in the central region, the difference was not statistically significant (Fig. 4c,d). These results suggest that Satb2-knockout cells are widely distributed in the transfected cortical area.

### Mutations in the *Satb2* gene induced by pX330-Satb2-2129 *in vivo*

**Figure 5:**
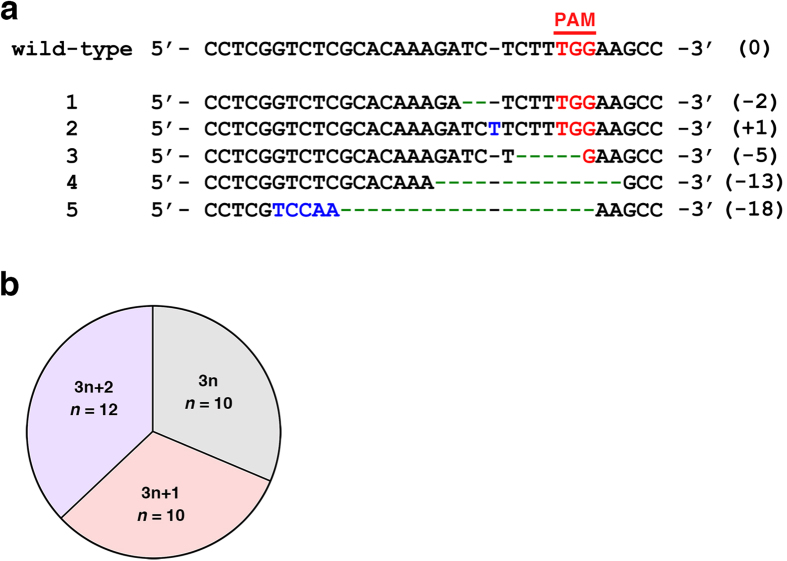
CRISPR/Cas9-mediated mutations in the *Satb2* locus. (**a**) Representative mutations found in the *Satb2* locus. The wild-type sequence of the *Satb2* gene and five representative mutations are shown. The PAM sequence is marked in red. Green dashes and blue bases indicate deletions and insertions, respectively. The numbers in parentheses indicate the number of bases that had been changed. (**b**) A pie chart showing frequencies of out-of-frame and of in-frame mutations found in the *Satb2* gene.

To investigate CRISPR/Cas9-induced mutations in the *Satb2* gene, we introduced 0.5 mg/ml pCAG-EGFP and 2.5 mg/ml pX330-Satb2-2129 into the developing mouse brain using *in utero* electroporation at E15.5. We extracted genomic DNA from the EGFP-positive area of the cerebral cortex at P4, PCR amplified the target site of the *Satb2* gene and then sequenced the PCR products using a next-generation sequencer. We successfully identified 40 kinds of indel mutations ranging from 1 bp to 291 bp in length in the target site of the *Satb2* gene (Fig. 5a). Of the 40 mutations identified, 8 mutations had relatively large deletions lacking the intron-exon junction, which lead to loss of function. The remaining 32 were indel mutations within the exon. Among these 32 mutations, 22 were out-of-frame indels (i.e., 3n + 1 bp or 3n + 2 bp in length) leading to the disruption of endogenous *Satb2* gene function, while 10 were in-frame mutations (Fig. 5b). These results suggest that delivery of pX330-Satb2-2129 using our procedure efficiently induces putative loss-of-function mutations in the *Satb2* gene *in vivo*.

### Introduction of pX330-Satb2-2129 recapitulated the phenotypes of *Satb2* knockout mice

Layer 2/3 neurons normally send their axons to the other side of the cerebral cortex through the corpus callosum. In *Satb2*^−/−^ mice, however, layer 2/3 neurons fail to send their axons to the other side of the cerebral cortex and instead extend their axons toward subcortical targets through the internal capsule^14^,^15^. Therefore, if the *Satb2* gene was knocked out by pX330-Satb2-2129, the projection patterns of layer 2/3 neurons should be affected.

**Figure 6:**
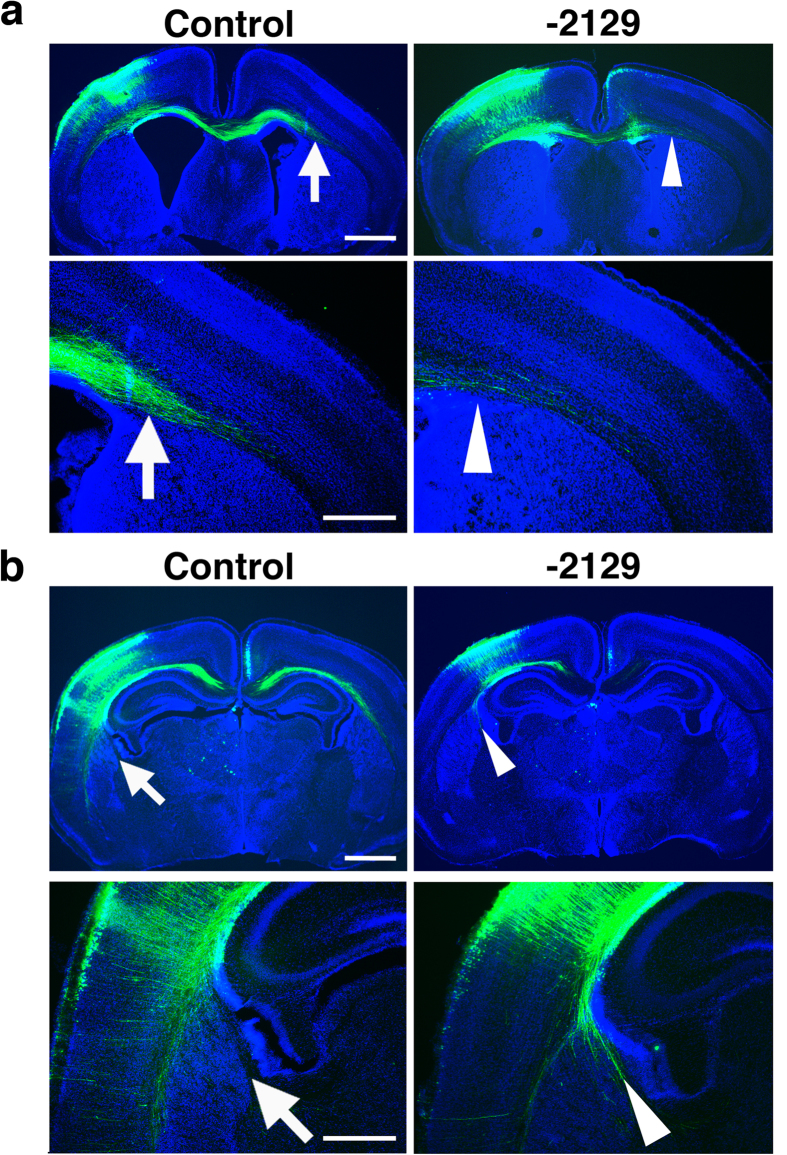
The effects of pX330-Satb2-2129 on axonal projection patterns of layer 2/3 neurons. Layer 2/3 neurons were co-transfected with pCAG-EGFP and pX330-Satb2 (2.5 mg/ml) using *in utero* electroporation at E15.5. Coronal sections were prepared at P4 and stained with anti-EGFP antibody (green) and Hoechst 33342 (blue). (**a**) Upper panels are low magnification images of coronal sections. Areas indicated by an arrow and an arrowhead in the upper panels are enlarged and shown in the lower panels. Note that EGFP-positive axons extended to the contralateral cortex in control animals (arrows), whereas they were markedly decreased by pX330-Satb2-2129 (arrowheads). Scale bars = 1 mm (upper) and 500 μm (lower). (**b**) Upper panels are low magnification images of coronal sections located more posterior to the sections in (**a**). Areas indicated by an arrow and an arrowhead in the upper panels are enlarged and shown in the lower panels. Note that no EGFP-positive axons were found in the internal capsule in control animals (arrows), whereas EGFP-positive axons were found in the internal capsule of animals transfected with pX330-Satb2-2129 (arrowheads). Scale bars = 1 mm (upper) and 500 μm (lower).

We transfected layer 2/3 neurons with 0.5 mg/ml pCAG-EGFP and 2.5 mg/ml pX330-Satb2-2129 using *in utero* electroporation at E15.5 and examined the projection patterns of EGFP-positive axons derived from transfected neurons^10^,^12^. As expected, in control animals transfected with the pX330 vector, EGFP-positive axons extended to the contralateral cortex (Fig. 6a, arrows), and no EGFP-positive axons were found in the internal capsule (Fig. 6b, arrows). In contrast, when layer 2/3 neurons were transfected with pX330-Satb2-2129, EGFP-positive axons were decreased in the contralateral cortex (Fig. 6a, arrowheads) and were found in the internal capsule (Fig. 6b, arrowheads). This phenotype is consistent with that observed in *Satb2*^−/−^ mice. Importantly, this phenotype is not observed in *Satb2*^+/−^ mice^14^. These results suggest that neurons transfected with pX330-Satb2-2129 carry biallelic mutations in the *Satb2* gene, resulting in alterations in their axonal projections.

### pX330-NeuN efficiently suppressed NeuN expression *in vivo*

**Figure 7:**
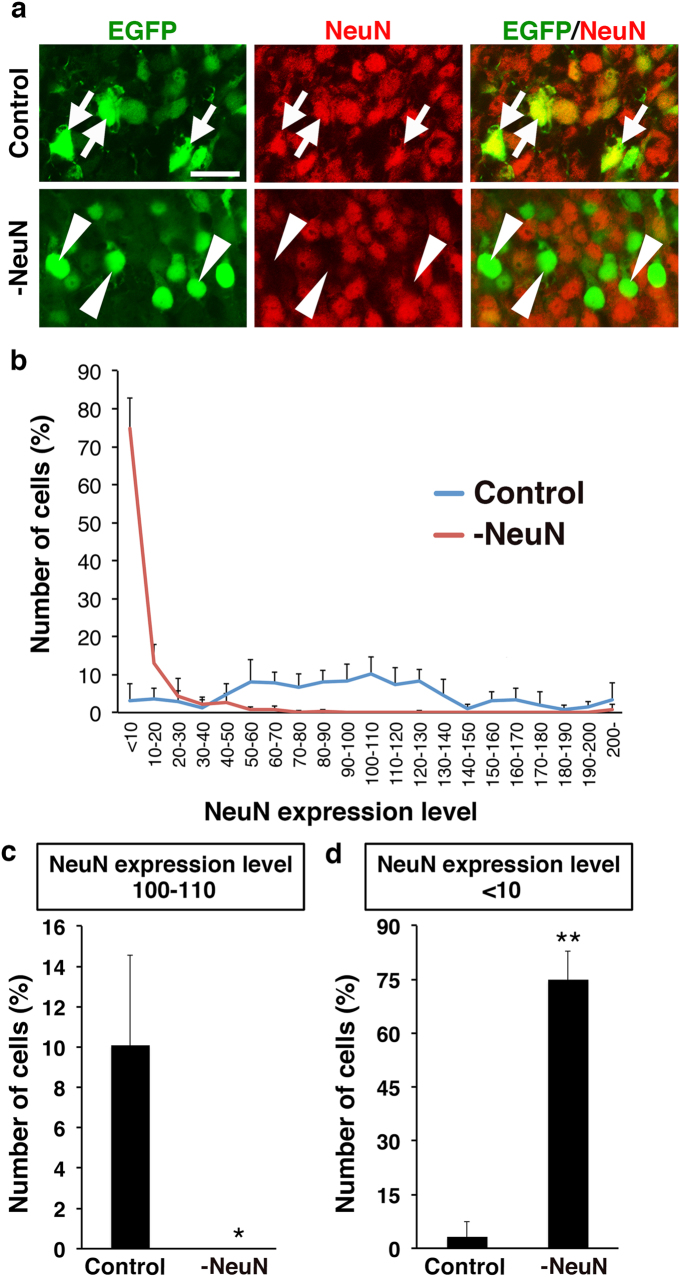
pX330-NeuN efficiently disrupts NeuN expression *in vivo*. (**a**) Layer 2/3 neurons were co-transfected with 0.5 mg/ml pCAG-EGFP and 2.5 mg/ml pX330-NeuN using *in utero* electroporation at E15.5. Coronal sections were prepared at P4 and immunostained with anti-NeuN antibody (red). Arrows indicate normal NeuN expression in EGFP-positive neurons transfected with a pX330 control plasmid. Arrowheads indicate a dramatic reduction of NeuN expression in EGFP-positive neurons transfected with pX330-NeuN. Scale bar = 20 μm. (**b**) Histogram of the expression levels of NeuN in transfected neurons. The average NeuN signal intensity in EGFP-positive neurons transfected with a pX330 control vector was set as NeuN expression level 100. The number of neurons with NeuN expression level 100-110 and that of neurons with NeuN expression level <10 are also shown in (**c,d**), respectively. (**c**) Neurons with normal levels of NeuN expression were eliminated by pX330-NeuN. *p < 0.05; Mann-Whitney U-test (*n* = 4 pups for each condition). Error bars indicate SD. (**d**) The number of neurons with no NeuN expression was greatly increased by pX330-NeuN. **p < 0.01; Welch’s *t*-test (*n* = 4 pups for each condition). Error bars indicate SD.

To test if our procedure is applicable to other genes, we constructed a pX330-NeuN plasmid, which contains an sgRNA targeting the neuron-specific RNA-splicing factor NeuN (Rbfox3)^19^. We co-transfected layer 2/3 neurons with 0.5 mg/ml pCAG-EGFP and 2.5 mg/ml pX330-NeuN using our procedure at E15.5. Consistent with our results for Satb2, NeuN immunoreactivity was strongly suppressed in the majority of EGFP-positive transfected neurons (Fig. 7a, arrowheads). Our quantitative analysis revealed that NeuN expression was lost in 75% of pX330-NeuN-transfected neurons (Fig. 7b,d) and that none of the remaining pX330-NeuN-transfected neurons had normal levels of NeuN expression (Fig. 7b,c). These results suggest that our procedure combining the CRISPR/Cas9 system and *in utero* electroporation is a powerful approach to achieve brain-specific gene knockout *in vivo*.

## Discussion

### Brain-specific gene knockout with the CRISPR/Cas9 system

Conditional gene knockout techniques are useful for investigating gene functions in tissue-specific and time-specific manners. Recently, the CRISPR/Cas9 system has been applied to the adult mouse brain using adeno-associated vial (AAV) vectors^19^,^20^. Swiech *et al*. designed a dual-vector AAV system that packages Cas9 and sgRNA expression cassettes in two separate AAV vectors, and demonstrated AAV-mediated genome editing in the adult mouse brain^20^. Platt *et al*. established a Cre-dependent Cas9 knockin mouse and demonstrated genome editing using AAV-mediated delivery of sgRNA in the adult mouse brain^19^. Although AAV-mediated CRISPR/Cas9 genome editing has been applied to reverse genetic studies of gene functions in the adult mouse brain, procedures for CRISPR/Cas9-mediated genome editing in the developing mouse brains have not been well established. In this study, we have demonstrated genome editing in post-mitotic neurons of the developing cortex with high efficiency by combining the CRISPR/Cas9 system and *in utero* electroporation. The introduction of pX330-Satb2-2129 induced disruption of Satb2 expression and resulted in a phenotype of axonal projections consistent with that previously observed in *Satb2* knockout mice. Although a recent pioneering study reported that gene knockout could be achieved by introducing CRISPR/Cas9 constructs using *in utero* electroporation^21^, quantitative information, such as the efficiency of gene knockout, was largely unavailable. Our findings indicate that the combination of the CRISPR/Cas9 system and *in utero* electroporation is a powerful and efficient approach to achieve gene inactivation in the living mouse brain.

Brain-specific gene knockout using the CRISPR/Cas9 system has several advantages over conditional mutagenesis using ES cells. The main advantage of the CRISPR/Cas9 system is that experimental procedures are by far easier and less time-consuming. In addition, the CRISPR/Cas9 system can easily induce disruptions of multiple genes by introducing a combination of pX330 plasmids. Furthermore, since *in utero* electroporation has been applied in gyrencephalic carnivore ferrets^22^,^23^, it seems plausible that our procedure can serve as a powerful technology for genomic perturbations in the brain of higher mammals.

Our data demonstrated that pX330-Satb2-2129 was most effective in disrupting the *Satb2* gene compared with pX330-Satb2-272 and -524 *in vivo* (Fig. 3). In contrast, all three constructs were similarly effective in inducing DSBs in reporter plasmids *in vitro* (Fig. 1). These results may indicate that the efficiency of inducing DSBs in reporter plasmids *in vitro* is not necessary always the same as that of gene knockout *in vivo*. Therefore, it seems appropriate to test several kinds of pX330 constructs in order to achieve effective gene knockout *in vivo*. A possible reason for the discrepancy between the data obtained with genomic DNA *in vivo* and reporter plasmids *in vitro* is that Cas9 has limited access to the target site of genomic DNA because genomic DNA is packaged into chromatin.

In addition, it should be noted that Satb2 immunoreactivity was still positive in 36% of pX330-Satb2-2129-transfected neurons (Fig. 4). There are several possible explanations for this result. The first possibility is that even if 36% of the transfected neurons were still Satb2-positive, some of them had CRISPR/Cas9-induced in-frame mutations. In fact, our sequence analysis showed that in-frame mutations were observed at a frequency of 25% in the *Satb2* gene (Fig. 5). In this case, Satb2 protein should be recognized by anti-Satb2 antibody even though it had mutations. It also seemed possible that the amount of pX330-Satb2-2129 plasmids was not large enough to induce biallelic mutations in the *Satb2* gene. This possibility is consistent with the fact that higher concentrations of pX330-Satb2-2129 more efficiently disrupted the *Satb2* gene (Figs 3 and 4). The third possibility is that the expression of Satb2 protein had already started when biallelic mutations were introduced in the *Satb2* gene.

### Satb2 is required for callosal axon projections cell-autonomously

Previous studies demonstrated that Satb2 functions as a regulatory determinant of callosal axon projections in the developing cortex. *Satb2* knockout mice showed an absence of callosal axons and an increase in subcortically projecting axons^14^,^15^. These results suggest that Satb2 is required for callosal axon projections. On the other hand, it was unclear whether Satb2 in layer 2/3 neurons is cell-autonomously crucial for callosal axon projections. It remained possible that Satb2 in other cell types was responsible for callosal axon projections of layer 2/3 neurons. In this study, we disrupted the *Satb2* gene in layer 2/3 neurons and found alterations in their axonal projections. Thus, our data strongly support the idea that Satb2 is required cell-autonomously for callosal axon projections. Our findings indicate that gene knockout in selected cell types using the CRISPR/Cas9 system is useful for distinguishing the cell-autonomous and non-cell-autonomous effects of loss-of-function mutations.

## Methods

### Animals

ICR mice were purchased from SLC (Hamamatsu, Japan) and were reared on a normal 12 h light/dark schedule. The day of conception and that of birth were counted as E0 and P0, respectively. All procedures were performed in accordance with protocols approved by the Kanazawa University Animal Care Committee.

### DNA constructs

pCAG-EGFP and pCAG-mCherry were described previously^12^. pX330, pX330-Cetn1, pCAG-EGxxFP and pCAG-EGxxFP-Cetn1 were obtained through Addgene. To construct pCAG-EGxxFP-Satb2, genomic fragments containing the sgRNA target sequence were PCR-amplified with forward and reverse primers containing *Nhe*I and *Eco*RI restriction sites, respectively. The amplified fragments were then digested by *Nhe*I and *Eco*RI, and cloned into the pCAG-EGxxFP plasmid digested with the same enzymes. Detailed primer information can be found in Supplementary Table S1. The pX330-Satb2 and pX330-NeuN plasmids expressing human Cas9 and sgRNA were prepared by ligating sgRNA oligonucleotides into the *Bbs*I site of pX330. Twenty-nucleotide sequences followed by the PAM sequence were used as seed sequences for sgRNA, and the sgRNA target sequences for Satb2 are shown in Fig. 1a. The sgRNA target sequence for NeuN was previously described^19^. If the first nucleotide was not G, we added an extra G at the 5′ end because the U6 promoter prefers a G for transcriptional initiation. Plasmids were purified using the EndoFree plasmid maxi kit (Qiagen, Germany).

### HEK293T cell transfection

One microgram of pX330-Satb2 was mixed with 1 μg of pCAG-EGxxFP-Satb2 and then introduced into HEK293T cells with polyethylenimine. To label transfected cells, 500 ng of pCAG-mCherry plasmid was used. The EGFP and mCherry fluorescence was observed under a fluorescence microscope 48 hours after transfection. To quantify the percentage of mCherry-positive transfected cells that became EGFP-positive, epifluorescence microscopic images were analyzed by ImageJ software (National Institutes of Health). Cells which had EGFP signal intensity stronger than the maximum signal intensity of the background in negative control samples were defined as EGFP-positive cells, and the number of EGFP-positive cells was counted. Statistical significance was determined using one-way analysis of variance.

### *In utero* electroporation

*In utero* electroporation was performed as described previously^7^,^10^,^12^,^24^. Briefly, at E15.5, pregnant ICR mice were anesthetized, and the uterine horns were exposed. Approximately 1–2 μl of DNA solution was injected into the lateral ventricle of embryos using a pulled glass micropipette. Each embryo within the uterus was placed between tweezer-type electrodes with a diameter of 3 mm (CUY650P3, NEPA Gene, Japan). Square electric pulses (45 V, 50 ms) were passed 5 times at 1 s intervals using an electroporator (ECM830, Harvard Apparatus, USA). Care was taken to quickly place embryos back into the abdominal cavity to avoid excessive temperature loss. The wall and skin of the abdominal cavity were sutured, and embryos were allowed to develop normally.

### Immunohistochemistry

Immunohistochemistry was performed as described previously with modifications^25^,^26^. Briefly, mice were anesthetized and transcardially perfused with 4% paraformaldehyde (PFA) in PBS. Dissected brains were post-fixed overnight with 4% PFA in PBS. To make coronal sections, the brains were cryoprotected by overnight immersion in 30% sucrose in PBS and embedded in OCT compound. Sections of 50 μm thickness were prepared using a cryostat, permeabilized with 0.3% Triton X-100 in PBS and blocked with 2% BSA and 0.3% Triton X-100 in PBS. The sections were incubated overnight with primary antibodies. After being incubated with Alexa 488- and Cy3-conjugated secondary antibodies and 1 μg/ml Hoechst 33342, the sections were washed and mounted. The primary antibodies included rabbit anti-GFP antibody (Medical & Biological Laboratories, Japan), mouse anti-Satb2 antibody (abcam) and rabbit anti-NeuN antibody (Cell Signaling).

### Microscopy

Epifluorescence microscopy was performed with a BZ-9000 microscope (KEYENCE, Japan). Confocal microscopy was performed with a FLUOVIEW FV10i (Olympus, Japan).

### Quantification of Satb2 and NeuN expression in the cerebral cortex

Confocal microscopic images were analyzed by ImageJ software (National Institutes of Health). After EGFP-positive nuclei were extracted by selecting Hoechst 33342 and EGFP double-positive areas, Satb2 signal intensities in EGFP-positive nuclei were measured. The Satb2 signal intensities were converted to “Satb2 expression level” values as follows. To normalize Satb2 signal intensities among samples, the average signal intensity of five Satb2-negative cells was set as 0, while the average signal intensity of five EGFP-negative non-transfected cells with the strongest Satb2 expression was set as 1, and the other values were converted linearly in each sample. Then, the average Satb2 signal intensity of the control brain was set as “Satb2 expression level 100”, and other values were converted linearly. Similar analyses were performed for the quantification of NeuN expression. Statistical significance was determined using Welch’s *t*-test or Mann-Whitney U-test.

### Next-Generation DNA Sequencing

EGFP-positive regions of the cerebral cortex transfected with pCAG-EGFP and pX330-Satb2-2129 were dissected under a fluorescent microscope, and genomic DNA was extracted using the DNeasy Blood & Tissue Kit (Qiagen, Germany). Genomic DNA including the sgRNA target site was PCR amplified using KOD FX DNA polymerase (TOYOBO, Japan) with the following primers: 5′-AGGCAGGGATTAAATTGCAGTGTAG-3′ (forward) and 5′-GGTGTGTTTGGGGAGATCCAG-3′ (reverse). The PCR amplicons were subjected to the construction of a library for Illumina sequencing with the KAPA Hyper Prep Kits for Illumina (Kapa Biosystems, Wilmington, MA). In brief, after being purified using AMPure XP beads (Beckman Coulter, Brea, CA), the PCR amplicons were subjected to end repair, A-tailing, adaptor ligation and PCR amplification for library construction with the KAPA Hyper Prep Kits for Illumina. The library was sequenced using an Illumina MiSeq sequencer (Illumina, San Diego, CA) with a 2 × 300 bp paired-end module. Our sequencing analysis yielded 2.6 million paired-end reads corresponding to 1.5 Gbp of valid sequence data without adapter sequences. When sequence overlap was found between each pair of paired-end reads, the read pair was merged, resulting in a read that would match a PCR product from which the paired-end reads were generated. After quality control, the high-quality merged reads were mapped to the target region of the mouse reference genome (*Mus musculus* C57BL/6 J chromosome 1 [GRCm38.p3 assembly, accession no. NC_000067.6]) using bwa 0.7.12^27^. The generated binary sequence alignment/map (bam) file was sorted and indexed using samtools 1.2^28^. Using the bam file, small indels were detected by HaplotypeCaller v.0.1.19 in GATK (GenomeAnalysisTK-3.3-0)^29^. Additionally, mapped reads with soft-clipping information were extracted from the bam file as sequence candidates with large indels. To detect large indels, the soft-clipped reads were realigned using a large gap assembly algorithm in Sequencher 5.1 (Gene Codes Corp., Ann Arbor, MI). The frequencies of reads with small/large indels were counted using an in-house developed script.

## Additional Information

**How to cite this article**: Shinmyo, Y. *et al*. CRISPR/Cas9-mediated gene knockout in the mouse brain using *in utero* electroporation. *Sci. Rep*. **6**, 20611; doi: 10.1038/srep20611 (2016).

## Supplementary Material

Supplementary Information
