# Supplementary material for: CRISPR/Cas9-mediated gene knockout in the mouse brain using in utero electroporation
Source: Sci Rep. 2016 Feb 9;6:20611. doi: 10.1038/srep20611 (PMC4746659; doi:10.1038/srep20611)
Supplement: Supplementary Information [file srep20611-s1.pdf]

Supplementary Information

CRISPR/Cas9-mediated gene knockout in the mouse brain using *in utero* electroporation

Yohei Shinmyo, Satoshi Tanaka, Shinichi Tsunoda, Kazuyoshi Hosomichi, Atsushi Tajima and Hiroshi Kawasaki

Supplementary Table S1. Primers used for constructing pCAG-EGxxFP-Satb2

Satb2-272 Fw	GCCGCTAGCTAAAGGCTTCGAGGTCCGACTCT
Satb2-272 Rv	GCCGAATTCACAGCGCCTAATCAACCTGAACC
Satb2-524 Fw	GCCGCTAGCTGGGCCACATGCTAAGATACCTC
Satb2-524 Rv	GCCGAATTCGTGCACATAAGCTCCCTGGGATT
Satb2-2129 Fw	GCCGCTAGCTTACCCGGCCCAGGTGACTCT
Satb2-2129 Rv	GCCGAATTCTCTTCCTCGGCCTCCACCTTGT
